# Cancer Immunology and Cancer Immunodiagnosis

**DOI:** 10.1155/2014/725691

**Published:** 2014-08-21

**Authors:** Jianying Zhang, Suxia Han, Bin Zhang, Yi Zhang

**Affiliations:** ^1^Cancer Autoimmunity Research Laboratory, Department of Biological Sciences, University of Texas, El Paso, TX 79968, USA; ^2^Department of Medical Oncology, The First Affiliated Hospital of Xi'an Jiao Tong University Medical Center, Shaanxi 710061, China; ^3^Division of Hematology/Oncology, Northwestern University Feinberg School of Medicine, Chicago, IL 60611, USA; ^4^Biotherapy Center and Department of Oncology, The First Affiliated Hospital, Zhengzhou University, Zhengzhou 450052, China

Cancer immunology is the study of interactions between the immune system and cancer cells, which is a rapid growing field of research that aims to identify biomarkers in cancer immunodiagnosis and to develop innovative cancer immunotherapeutic strategies. The immune response, including the recognition of cancer-specific antigens, is of particular interest in cancer immunology field, which can further drive the development of new vaccines and antibody therapies. It is also well demonstrated that the immune system can recognize the antigenic changes in cancer cell and further develop antibody against these cellular antigens that have been generally called tumor-associated antigens (TAAs) [1–4]. These cancer-associated anti-TAAs autoantibodies might be considered as “reporters” from the immune system, to identify the antigenic changes in cellular proteins involved in the transformation process [5, 6]. There has been a growing interest in using serum autoantibodies against TAAs as biomarkers in cancer immunodiagnosis. The persistence and stability of these antibodies in the serum samples of cancer patients is an advantage over other potential markers, including the TAAs themselves, some of which are released by tumors but are rapidly degraded or cleared after circulating in the serum for a limited time [7]. In recent years, the potential utility of TAA-autoantibody systems as early cancer biomarker tools to monitor therapeutic outcomes or as indicators of disease prognosis has been explored.

Activation of the immune system for therapeutic benefit in cancer has long been a goal in immunology and oncology. The passive cancer immunotherapy has been well established for several decades, and continued advances in antibody and T-cell engineering should further enhance their clinical impact in the years to come. In contrast to these passive immunotherapy strategies, the active cancer immunotherapy has been proved elusive. In the context of advances in the understanding of how tolerance, immunity, and immunosuppression regulate antitumour immune responses together with the advent of targeted therapies, these successes suggest that active immunotherapy represents a path to obtain a durable and long-lasting response in cancer patients [8]. The key to cancer immunodiagnosis and immunotherapy is an improved understanding of the immune response during malignant transformation.

According to this background, we have invited investigators to contribute original research articles as well as review articles describing cancer immunodiagnosis and cancer immunotherapy and assembled this special issue for updating the recent advances in this field. In this special issue, we have included a total of 18 papers including 12 original research papers and 6 review papers, in which 7 research papers deal with cancer immunotherapy and 5 research papers deal with cancer immunodiagnosis. For example, a paper of Z. B. Wu et al. has demonstrated that glioma-associated antigen HEATR1 can induce functional cytotoxic T lymphocytes in patients with glioma; a paper of S. I. Kim et al. has discussed the impact of underweight after treatment on prognosis of advanced-stage ovarian cancer; a paper of J. Ma et al. has indicated that the intensity of radiotherapy-elicited immune response is associated with esophageal cancer clearance; a paper of J. Li et al. has discussed the selective depletion of regulatory T cell subsets by docetaxel treatment in patients with non-small cell lung cancer. Papers from P. Wang et al., L. Wang et al., L. Chen et al., L. Borska et al., and J. Gu et al. have, respectively, discussed different cancer-associated protein biomarkers in cancer immunodiagnosis and cancer prognosis. In addition, review papers cover many aspects relating to cancer immunotherapy and cancer immunodiagnosis. For example, a review paper of Palacios-Arreola MI et al. has discussed the role of chemokines in breast cancer pathology and its possible use as therapeutic targets; a paper of J. Lacombe et al. has discussed the use of autoantibodies in detection of breast cancer; a paper of D.-S. Chung et al. has discussed a new hope of immunotherapy for malignant gliomas; a paper of D. A. Erkes and S. R. Selvan has extensively reviewed the hapten-induced contact hypersensitivity, autoimmune reactions, and tumor regression.

In summary, this special issue covers many important aspects in cancer immunology, including recent advances in the identification and evaluation of TAA and anti-TAA biomarkers in cancer immunodiagnosis, as well as the basic and clinical studies relating to cancer immunotherapy. We hope that this special issue can provide some useful information to investigators in the field of cancer immunodiagnosis and cancer immunotherapy and also give the readers a sense of some of the advancements made in this field.

*Jianying Zhang* *Jianying Zhang* *Suxia Han* *Suxia Han* *Bin Zhang* *Bin Zhang* *Yi Zhang* *Yi Zhang*
